# Performance of European and American Societies of Gastrointestinal Endoscopy Guidelines for Prediction of Choledocholithiasis in Patients with Acute Biliary Pancreatitis

**DOI:** 10.3390/medicina59122176

**Published:** 2023-12-15

**Authors:** Žan Peter Černe, Nejc Sever, Luka Strniša, Samo Plut, Jan Drnovšek, Jurij Hanžel, Darko Siuka, Borut Štabuc, David Drobne

**Affiliations:** 1 Faculty of Medicine, University of Ljubljana, 1000 Ljubljana, Slovenia; zan.cerne@gmail.com (Ž.P.Č.); jan.drnovsek@kclj.si (J.D.); borut.stabuc@kclj.si (B.Š.); 2 Department of Gastroenterology, University Medical Centre Ljubljana, Japljeva 2, 1000 Ljubljana, Slovenia; nejc.sever@kclj.si (N.S.); luka.strnisa@kclj.si (L.S.); samo.plut@kclj.si (S.P.); jurij.hanzel@kclj.si (J.H.); darko.siuka@kclj.si (D.S.)

**Keywords:** acute biliary pancreatitis, endoscopic ultrasound, ERCP, choledocholithiasis, ultrasound, transabdominal ultrasound

## Abstract

*Background and Objectives:* Up to one-third of patients with acute biliary pancreatitis also present with choledocholithiasis. Guidelines from the European Society of Gastrointestinal Endoscopy (ESGE) and the American Society for Gastrointestinal Endoscopy (ASGE) for investigating suspected choledocholithiasis suggest endoscopic retrograde cholangiopancreatography in patients with high-likelihood (ESGE)/high-probability (ASGE) predictors and endoscopic ultrasound in those with intermediate-likelihood (ESGE)/intermediate-probability (ASGE) predictors. Although both guidelines are similar, they are not identical. Furthermore, these algorithms were mainly developed from cohorts of patients without pancreatitis and are therefore poorly validated in a subset of patients with acute pancreatitis. We aimed to assess the performance of the ESGE and ASGE algorithms for the prediction of choledocholithiasis in patients with acute biliary pancreatitis. *Materials and Methods:* This was a retrospective analysis of 86 consecutive patients admitted to a tertiary referral centre in the year 2020 due to acute biliary pancreatitis. *Results:* Choledocholithiasis was confirmed in 29/86 (33.7%) of patients (13 with endoscopic retrograde cholangiopancreatography and 16 with endoscopic ultrasound). All 10/10 (100%) ESGE high-likelihood and 14/19 (73.7%) ASGE high-probability patients had choledocholithiasis. Only 19/71 (26.8%) patients with ESGE intermediate likelihood and 15/67 (22.4%) with ASGE intermediate probability had choledocholithiasis. Only 8/13 (61.5%) patients with the ASGE high-probability predictor of dilated common bile duct plus bilirubin > 68.4 µmol/mL had choledocholithiasis. Since this predictor is not considered high likelihood by ESGE, this resulted in a superior specificity of the European compared to the American guideline (100% vs. 91.2%). Following the American instead of the European guidelines would have resulted in five unnecessary endoscopic retrograde cholangiopancreatographies and five unnecessary endoscopic ultrasound examinations. *Conclusions:* This retrospective analysis suggests that the European guidelines may perform better than the American guidelines at predicting choledocholithiasis in the setting of acute pancreatitis. This was because dilated common bile duct plus bilirubin > 68.4 µmol/mL was not a reliable predictor for persistent bile duct stones.

## 1. Introduction

The incidence of acute biliary pancreatitis (ABP) has been steadily increasing throughout most European countries in the past decades [1,2,3]. Although the pathogenesis of ABP is multifaceted, the key feature is ampullary obstruction by gallstones [4,5]. Fortunately, most offending stones spontaneously migrate into the duodenum [5,6,7]. However, in up to one-third of patients, the stones do not pass spontaneously from the common bile duct (CBD) to the duodenum [6,8]. These patients should be recognized and the CBD cleared by endoscopic retrograde cholangiopancreatography (ERCP) to relieve obstruction and prevent further complications. Clinicians often find it difficult to accurately identify this subset of patients with non-invasive modalities such as transabdominal ultrasound (US) or computerized tomography (CT), as these have somewhat low sensitivity (50–80% and 40%, respectively) [9]. The strategy of performing ERCP in all patients suspected to have CBD stones based on clinical, biochemical, and imaging techniques (transabdominal US or CT) would result in a high proportion of ERCP examinations with no stones found in the CBD. As ERCP has a significant risk of complications, such diagnostic ERCP should be avoided [10]. Magnetic resonance cholangiopancreatography (MRCP) provides high sensitivity and specificity for gallstones in ABP, but its availability is still very limited, especially in the emergency setting [9,11]. Due to these facts, either endoscopic ultrasound (EUS) or ERCP are performed to assess the CBD for pathology. Since EUS and ERCP are expensive and invasive procedures, with the latter also carrying a risk of serious complications, there is a need for a tool to help better stratify patients who require EUS or ERCP [9,12,13,14,15,16,17].

Up until 2019, clinicians relied on the American Society for Gastrointestinal Endoscopy (ASGE) 2010 guidelines on the role of endoscopy in the evaluation of suspected choledocholithiasis which classified clinical gallstone pancreatitis as a “moderate” predictor; thus, all patients with ABP were classified to have at least an intermediate probability for choledocholithiasis and were offered an EUS, provided the availability of local expertise and coverage by insurance [18]. However, in 2019, after reviewing new studies, the ASGE removed ABP from its guidelines as a predictor for choledocholithiasis [8,19,20,21]. This led to some uncertainty concerning the approach towards these patients. Despite ABP being removed from the 2019 ASGE guidelines, most clinicians still rely on these guidelines, as specific guidelines for patients with ABP are not available [19,22]. These guidelines suggest ERCP for patients with any high-probability predictor for choledocholithiasis (CBD stones on transabdominal US/cross-sectional imaging, signs of ascending cholangitis, or total bilirubin > 68.4 µmol/L plus dilated CBD on transabdominal US/cross-sectional imaging) or EUS for patients with any intermediate-probability predictor (dilated CBD on transabdominal US/cross-sectional imaging, pathologic liver tests or age over 55 years) [19]. Similar, but not identical, guidelines on this have also been published by the European Society of Gastrointestinal Endoscopy (ESGE) in 2019 [22]. An important difference between the two guidelines is that the ESGE guidelines suggest immediate ERCP only in patients with features of cholangitis or CBD stones identified on a transabdominal US, but not in patients with abnormal liver function tests plus dilated CBD on a transabdominal US as is the case with the ASGE 2019 guidelines [19,22].

However, there is one caveat—it is difficult to apply the ASGE 2019 or ESGE guidelines to patients with ABP as these have not been validated in patients with ABP, but are generally intended for patients with suspected choledocholithiasis without pancreatitis [19,22]. Pathogenesis in ABP may directly interfere with the predictors used by both guidelines and influence their reliability [4,19,22]. For example, spontaneous passage of a gallstone or sludge can cause oedema and spasms of the ampulla of Vater, resulting in biliary obstruction, which can present as a moderate elevation of bilirubin and dilation of the CBD despite the absence of choledocholithiasis [13,23]. Moreover, pancreatitis can cause high fever due to systemic inflammatory response in the absence of bacterial infection [24]. High fever in such patients can be misinterpreted as a sign of cholangitis, leading to unnecessary ERCP [13,15,19,22,25]. The sonographic visibility of the upper abdomen is typically reduced in patients with ABP due to bowel paresis, further decreasing the reliability of transabdominal US for assessing distal CBD [26]. Consequently, the utility of transabdominal US to assess two high-probability ASGE predictors (the presence of a CBD stone and CBD dilation plus elevated levels of bilirubin) and one high-likelihood ESGE predictor (the presence of a CBD stone) is decreased in the settings of pancreatitis [4,19,22,26].

Due to these limitations, our institution has a low threshold for EUS before ERCP in patients with ABP, as similarly suggested by the ESGE guidelines [19,22]. The global aim of this report was to evaluate if such a strategy is justified. Our specific aim was to assess the performance of both the ASGE and ESGE guidelines for the prediction of choledocholithiasis in patients with ABP.

## 2. Materials and Methods

**Figure 1:**
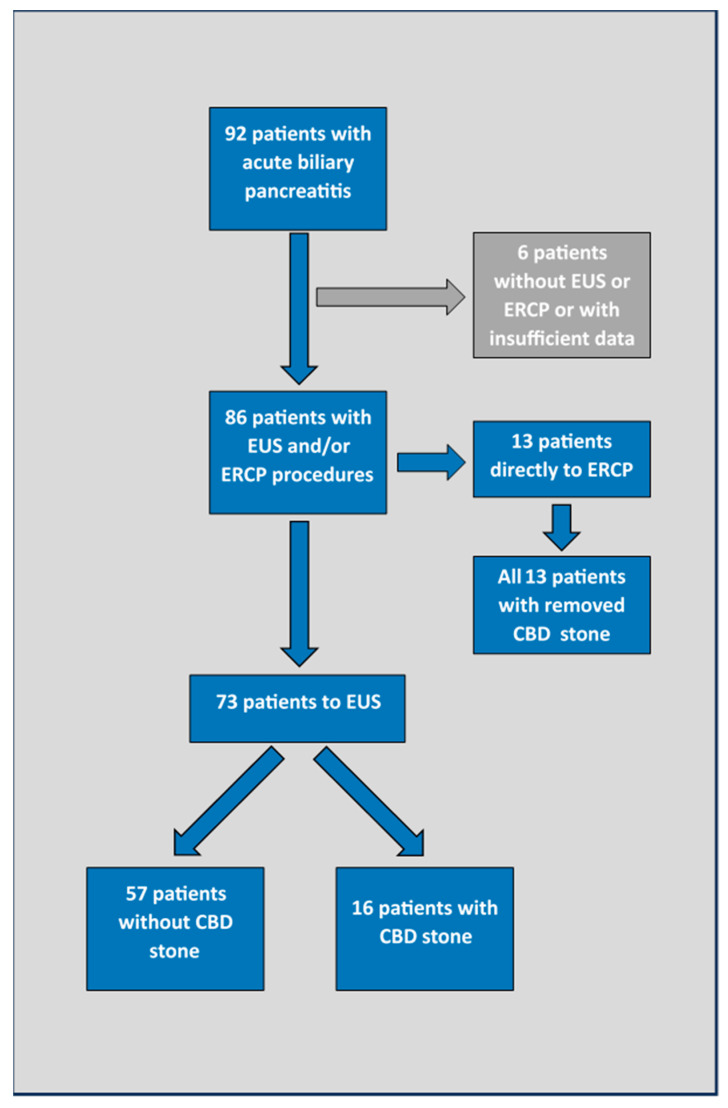
Patient flow. EUS—endoscopic ultrasound; ERCP—endoscopic retrograde cholangiopancreatography; CBD—common bile duct.

In this retrospective cohort study, we included all patients admitted to the tertiary referral gastroenterology centre due to ABP in the year 2020. Using an electronic database, we identified 92 patients hospitalized for ABP in this period. Only 6 out of 92 (6.5%) were not assessed for the presence of CBD stones by EUS or ERCP, or had insufficient laboratory data and were therefore excluded from further analysis. Patient flow is depicted in Figure 1.

The diagnosis of ABP was made in accordance with the revised Atlanta classification system [27].

The diagnosis of ABP was based on the finding of stones in the gallbladder or in the CBD, in combination with typical biochemical results after excluding other aetiologies [4].

The diagnosis of concomitant acute cholangitis was made at the discretion of the treating physician (an experienced gastroenterologist), in line with the Tokyo 2018 criteria [28], but also relying on indicators of bacterial infection such as elevated serum procalcitonin levels and positive blood cultures. As per local clinical practices, an EUS was performed on all patients with ABP apart from patients with clinical and laboratory signs of cholangitis or choledocholithiasis clearly visualized on the transabdominal US or CT. Patients with the latter two criteria were referred directly to ERCP. Exceptionally, patients proceeded directly to ERCP based on the decision made by the treating gastroenterologist.

Thus, EUS was performed on all patients with the intermediate-probability ASGE and intermediate-likelihood ESGE predictors for choledocholithiasis [19,22]. Importantly, all patients with the ASGE high-probability predictor of a total bilirubin > 68.4 µmol/L plus dilated CBD on their transabdominal US were also referred to EUS before potential ERCP (contrary to the ASGE 2019 guideline) [19].

The transabdominal US was performed at the emergency department by a radiologist. The common bile duct was considered dilated when wider than 6 mm, or in the case of prior cholecystectomy, wider than 8 mm, in line with previous definitions [8,20,21,29,30]. A common bile duct stone was defined as a hyperechogenic finding with the diameter ≥ 3mm, with dorsal acoustic shadow (transabdominal US and EUS), or stone visualization during ERCP [19]. Endoscopic ultrasound procedures were performed by 5 different gastroenterologists with a total annual volume of around 1300 procedures, using a Fujifilm EG-580 UR endoscope, and 5 different gastroenterologists with a total annual volume of around 1400 procedures performed ERCP.

All patients had standard biochemical tests performed at the emergency department. A subset of patients had repeated biochemical tests just before EUS/ERCP, typically the morning after their admission to the ward. Liver biochemical tests were considered abnormal if at least one of the parameters (total or direct bilirubin, transaminases, or alkaline phosphatase) was >2× above the upper normal level [19,22].

The reliability of the ASGE and ESGE predictors (Table 1) for CBD stones was assessed by calculating the sensitivity and specificity of each predictor. In a subset of patients with available paired biochemical measurements, we also evaluated whether the dynamics of liver biochemistry could predict spontaneous passage of CBD stones.

### Statistical Analysis

For the statistical analysis we used Mann–Whitney U, Chi square/Fisher’s exact tests and receiver operating characteristic (ROC) curve analysis with Youden’s J statistics [31]. A *p*-value < 0.05 was considered statistically significant. Reported *p* values are nominal unless otherwise stated. Analyses were performed using IBM SPSS (Version 22) and GraphPad Prism (Version 8). Ethical approval was obtained from the Slovenian Commission for Medical Ethics (application number 0120-486/2022/3).

## 3. Results

A total of 86 out of 92 (93.5%) patients who were admitted in 2020 due to ABP were included in this analysis (Figure 1).

Patients’ characteristics, duration of symptoms relative to examinations performed, and laboratory values are summarized in Table 2.

### 3.1. Endoscopic Ultrasound and Endoscopic Retrograde Cholangiopancreatography Findings

Common bile duct stones were confirmed in all 13 patients who were directly referred to ERCP. Ten of these had cholangitis, a CBD stone visualized on the transabdominal US, or both criteria. Three patients were referred to ERCP based on clinical decisions (one patient with a rapid increase in serum bilirubin and two older, fragile patients not suitable for cholecystectomy where endoscopic biliary sphincterotomy was considered to be the definite treatment).

All other patients (73/86 (84.9%)) underwent EUS after a median time of 2 days (interquartile range 1–3 days) from their first presentation at the emergency department. EUS eventually confirmed CBD stones in 16/73 (21.9%) of these patients. In one patient, the EUS detected a very small fragment with a diameter of only 3 mm located in the distal part of the CBD. In this patient, ERCP was not performed (clinical decision) and a second EUS a few days later confirmed spontaneous passage.

### 3.2. Performance of American Society for Gastrointestinal Endoscopy 2019 Guidelines and European Society of Gastrointestinal Endoscopy Guidelines for Prediction of Choledocholithiasis in Patients with Acute Biliary Pancreatitis

Predictors used in the ASGE and ESGE guidelines are summarised in Table 1. Table 3 shows the proportion of patients with an eventually confirmed CBD stone according to the presence of different ASGE and ESGE predictors. In Table 4, the high-, intermediate-, and low-probability predictors of ASGE, and the high-, intermediate-, and low-likelihood ESGE predictors are compared.

The sensitivity and specificity of each predictor and risk group of the ASGE and ESGE guidelines are depicted in Table 5.

### 3.3. Predictive Values of Liver Biochemistry for the Presence of Common Bile Duct Stones in Patients with Acute Biliary Pancreatitis

**Figure 2:**
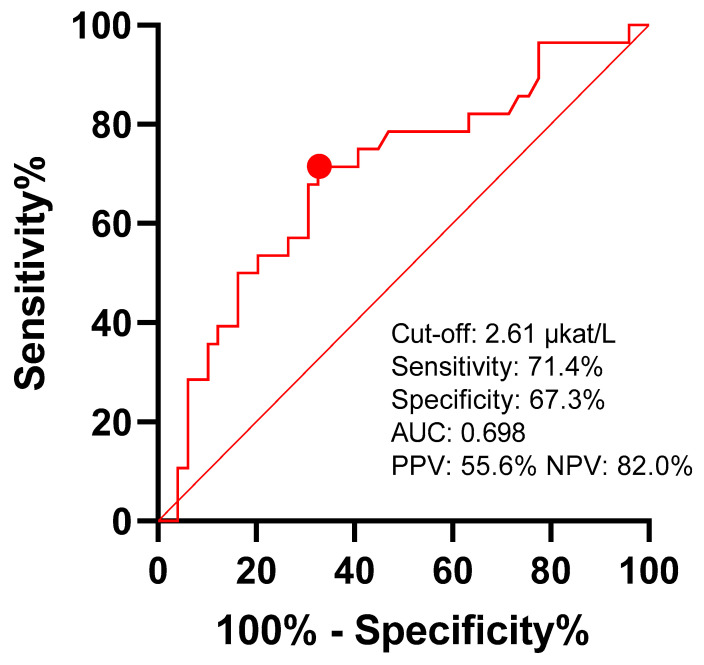
Predictive value of alkaline phosphatase for choledocholithiasis. Youden’s J statistics cut-off point is marked with red dot. AUC—area under curve; PPV—positive predictive value; NPV—negative predictive value.

Liver biochemistry at admission (Table 6) was not associated with the presence of CBD stones as patients with and without choledocholithiasis had similar values. Only the alkaline phosphatase values were slightly higher in patients who later had confirmed CBD stones (area under the ROC curve of 69.8% (95% confidence interval: 57.3–82.3%, *p* = 0.004)) (Figure 2) [31].

**Figure 3:**
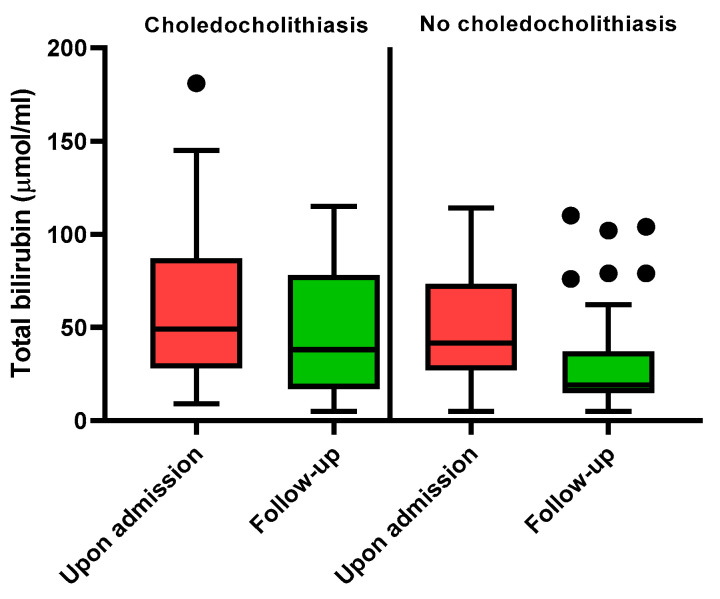
Bilirubin levels in patients with and without choledocholithiasis.

Similarly, by analysing the data of 70/87 (80.5%) patients who had available paired measurements of biochemistry (baseline at the emergency department and follow-up value after admission to the ward; the time span between both measurements was median 1 day (interquartile range: 1–3 days)), we did not find any predictive value of follow-up biochemistry for choledocholithiasis. Furthermore, the dynamics of biochemistry were similar in patients with or without CBD stones, apart from one patient, whose total bilirubin levels rose from 33 to 115 µmol/L and was consequently referred directly to ERCP that confirmed choledocholithiasis. The dynamics of total bilirubin are depicted in Figure 3.

## 4. Discussion

The removal of persistent CBD stones is important in the management of ABP [22,32,33,34,35,36]. Since ERCP can have serious, potentially life-threatening complications, every effort is made to confirm the presence of stones in the CBD before ERCP [13,19,22]. The ASGE and ESGE guidelines suggest using algorithms that stratify patients with suspected CBD stones in three groups: low, intermediate, and high probability/likelihood. However, these guidelines were mainly developed from cohorts of patients without pancreatitis [19,22]. Furthermore, the presence of ABP was historically used as a variable in patients with suspected choledocholithiasis, mainly decreasing the chance of CBD stone persistence [8,19,20,21]. However, the performance of predictors was scarcely tested within the subgroup of patients with ABP [29].

Because of specific pathological changes in ABP that may impact the sensitivity of transabdominal US and influence liver biochemistry, which are the two input variables central to the ASGE and ESGE algorithms for predicting CBD stones, it is important to validate the guidelines in this subset of patients [4,19,22]. We did this in our retrospective cohort study. Our findings confirmed that the high-probability/likelihood predictors (visualized stones in the CBD by transabdominal US and features of cholangitis) correctly identified patients with persistent CBD stones who can bypass EUS and proceed directly to ERCP. Conversely, the third high-probability ASGE predictor for choledocholithiasis, total bilirubin > 68.4 µmol/L plus dilated CBD, was only correct in predicting CBD stones in half of the patients with ABP. The value of this predictor was numerically similar to the intermediate-probability ASGE predictors (age > 55 years, dilated CBD, and abnormal liver function tests) and intermediate-likelihood ESGE predictors (abnormal liver function tests and dilated CBD). We speculate that one of the reasons for this deviation might be oedema and spasms of the ampulla of Vater after the spontaneous passage of CBD stones resulting in the transient elevation of bilirubin and CBD dilation [4,19,23]. Strictly following the ASGE algorithm in our cohort would have resulted in five patients undergoing unnecessary ERCP. We therefore suggest, that in the setting of ABP, patients with elevated bilirubin and dilated CBD are first referred to EUS rather than directly to ERCP. However, it should be noted that we did not address the timing of ERCP in our cohort. This is relevant since early ERCP with sphincterotomy did not change the outcome of patients with predicted severe pancreatitis and nor did EUS-guided ERCP [37,38].

Applying the ESGE algorithm to our cohort resulted in five patients being classified as low likelihood. These five patients were classified as intermediate probability by the ASGE 2019 guidelines as they were older than 55 years. None of them had choledocholithiasis. If the ESGE guidelines were followed, these patients could have been spared an EUS. Additionally, since all patients with choledocholithiasis showed dilated CBDs on the transabdominal US or abnormal liver biochemical tests, there was no additional benefit in using the third ASGE intermediate-probability predictor (age over 55) in our cohort.

Normal liver biochemistry has a high negative predictive value for choledocholithiasis [18,19,22]. However, since most patients in our cohort had increased liver function tests this was not useful for excluding CBD stones. Our analysis also did not reveal useful differences in the levels of liver function tests (total and direct bilirubin, alkaline phosphatase, and aspartate and alanine aminotransferase) between patients with or without CBD stones. Alkaline phosphatase had the highest predictive value among biochemical tests, but the area under ROC was still below 70%, thus precluding its clinical utility. The predictive value of liver function tests also did not improve after incorporating the secondary laboratory set after 1–3 days from presentation. The same conclusions have been previously published by other authors. Repeating these tests before deciding to proceed with either EUS or ERCP appears not to be clinically useful [8,39]. The only test that may be worth repeating is total bilirubin, since its doubling to >68.4 µmol/L could be a sign of persistent CBD obstruction by a gallstone [30]. However, this occurred in only one of our patients and the reported positive predictive values from He and colleagues (62%) do not justify the immediate use of ERCP in these patients [30].

A dilated CBD was the most reliable intermediate-probability/likelihood predictor of choledocholithiasis in our cohort, which is in line with previous studies that were not exclusively performed in patients with ABP [20,21,30]. However, the sensitivity of CBD dilatation for choledocholithiasis was lower in our patients with ABP (55.2%) compared to the sensitivity reported in studies that predominantly included patients without pancreatitis (from 58% to 83.8%) [20,21,30]. This could be due to the aforementioned decreased performance of transabdominal US caused by bowel paresis in the setting of acute pancreatitis (26). Alternatively, patients with ABP may be more likely to have small and distally located stones, which are more difficult to find with transabdominal US [6,30].

Our study was an open-label cohort study with all the potential intrinsic limitations. Despite its retrospective design, we still believe we largely avoided selection bias since we analysed all patients admitted in the year 2020 to a large tertiary referral centre covering a predefined urban area. Also, the majority of patients were systematically approached for CBD stones in a similar way, with a low threshold for EUS examination (following ESGE guidelines) [22]. The literature has shown that EUS is more sensitive compared to MRCP in the detection of very small stones and sludge [9,11]. Therefore, we might have more accurately assessed choledocholithiasis compared to some other studies that used the latter two modalities as the golden standard [29,30]. However, very small stones or sludge, that could potentially be missed with MRCP, might not be a clinically important finding in ABP. Nevertheless, performing EUS instead of MRCP might be more practical in some institutions as in the case of CBD stones where the patient can have ERCP performed during the same session.

In the setting of acute pancreatitis, it is challenging to reliably establish the diagnosis of concomitant acute cholangitis. Using the Tokyo 2018 guidelines for acute cholangitis [28], virtually all the criteria can also be observed in acute pancreatitis due to pathophysiological, non-infectious processes ongoing during pancreatitis. Nevertheless, the diagnosis of concomitant acute cholangitis in our study was not only made at the discretion of the treating physician based on the Tokyo guidelines, but also on the indicators of bacterial infection. Despite this somewhat arbitrary approach, the strategy proved to be a highly accurate predictor of persistent CBD stones in our cohort. Further studies are needed to validate a tool that would promptly discern cholangitis in patients with ABP.

The APEC trials failed to demonstrate a difference for the primary outcome, which was a composite of mortality or major complication occurring within 6 months of randomization. The only component of the primary outcome with a difference between groups was cholangitis, which was less common in the urgent ERCP group than in the conservative treatment group (2% versus 10%) [37,38]. The findings of the APEC studies, in conjunction with prior work, emphasize that expecting ERCP to stop or reverse the pathophysiological mechanisms resulting in systemic and local complications in acute pancreatitis is overly optimistic. Biliary obstruction is removed with ERCP, which is reflected by the lower incidence of cholangitis in the urgent ERCP group but does not impact the development of organ failure or pancreatic necrosis, the driving mechanisms of mortality in acute pancreatitis. Hypothetically, an even earlier intervention could conceivably alter the disease course, but the intervention (either urgent ERCP or EUS to assess the need for ERCP) was performed within 24 h of presentation, with a quarter of ERCP performed within 12 h in APEC-1 [38], which is probably already at the limit of feasibility in most, if not all, healthcare settings.

In this study, the data on the complications following EUS or ERCP were not collected. However, we believe that our approach with early EUS was safe as the safety of EUS is well documented [40,41]. To the contrary, ERCP may be associated with significant procedure-related complications [42]. Here it should also be acknowledged that our approach with early EUS might not have always been justified since the EUS-guided ERCP approach, even in predicted severe pancreatitis, did not reduce major complications or mortality compared to the conservative strategy in APEC trials [37,38]. This might be even more relevant in predicted mild pancreatitis. Unfortunately, we were unable to perform more focused analyses (stratified by predicted severity), since retrospectively assessing predicted disease severity would be unreliable from clinical charts.

## 5. Conclusions

In conclusion, our retrospective analysis of patients with ABP suggests that the ESGE 2019 high-likelihood predictors for CBD stone prediction perform better than the ASGE 2019 high-probability predictors in the setting of ABP. The difference can be attributed to the fact that half of the patients with the ASGE 2019 predictor of dilated CBD plus bilirubin above > 68.4 µmol/mL did not have CBD stones on their EUS. Our observation might be specific to a subgroup of patients with ABP and needs further exploration in larger cohorts to help prevent unnecessary ERCP procedures. Future guidelines should separately address the assessment of the CBD for persistent stones in a subgroup of patients with ABP.

## Figures and Tables

**Table 1:** American society for Gastrointestinal Endoscopy 2019 and European society of Gastrointestinal Endoscopy algorithms for suspected common bile duct stones.

American society for Gastrointestinal Endoscopy (ASGE) 2019 guideline—probability of common bile duct stones	
High	Common bile duct stone on transabdominal US/cross-sectional imaging
or	Clinical ascending cholangitis
or	Total bilirubin > 68.4 µmol/mL (>4 mg/dL) and dilated common bile duct on transabdominal US/cross-sectional imaging
Intermediate	Abnormal liver biochemical tests *
or	Age > 55 years
or	Dilated common bile duct on transabdominal US/cross-sectional imaging
Low	No predictors present
European Society of Gastrointestinal Endoscopy (ESGE)—likelihood of common bile duct stone	
High	Features of cholangitis
or	Common bile duct stones on transabdominal US
Intermediate	Abnormal liver function tests *
and/or	Common bile dilatation on transabdominal US
Low	Normal liver function tests and no common bile duct dilatation on transabdominal US
Terminology by American Society for Gastrointestinal Endoscopy 2019 and European Society of Gastrointestinal Endoscopy 2019 original papers.	

* no specific cut-off value defined in either guideline.

**Table 2:** Patients’ evaluation details before endoscopic ultrasound examination.

	Value (Interquartile Range)
Number of patients, (n)	86
Age at admission (years)	66.0 (48.8–78.3)
Gender, female/male [%]	62/38
Total hospital stay length, (days)	8.0 (6.0–12.0)
Time from admission to EUS (days)	2.0 (1.0–3.0)
Time from EUS to ERCP (days)	0 (0–1.8)
Time from admission to ERCP (if only ERCP was performed) (days)	1.0 (1.0–2.0)
Transabdominal ultrasound findings (n = 82)	
Cholecystolithiasis on transabdominal US	59 (72.0%)
Sludge in gallbladder on transabdominal US	6 (7.3%)
Previous cholecystectomy	7 (8.5%)
Empty gallbladder on transabdominal US	9 (11.0%)
No data on gallbladder examination	1 (1.2%)
Common bile duct dilation	27 (32.9%)
Liver biochemistry	
At emergency department ^1^	
Total serum bilirubin (µmol/L) n = 82	46.0 (27.0–84.0)
Direct serum bilirubin (µmol/L) n = 80	28.5 (14.0–52.0)
Alkaline phosphatase (µkat/L) n = 77	2.5 (1.8–4.6)
Aspartate aminotransferase (µkat/L) n = 85	4.5 (2.3–7.0)
Alanine aminotransferase (µkat/L) n = 85	5.8 (2.78–9.7)
Control laboratory ^2^	
Total serum bilirubin (µmol/L) n = 68	21.5 (15.4–59.0)
Direct serum bilirubin (µmol/L) n = 68	12.0 (6.0–35.0)
Alkaline phosphatase (µkat/L) n = 68	2.2 (1.4–3.3)
Aspartate aminotransferase (µkat/L) n = 68	1.4 (0.7–3.0)
Alanine aminotransferase (µkat/L) n = 68	2.9 (1.6–5.4)

^1^ data available for 85/86 patients, data was excluded pairwise. ^2^ data available for 69/86 patients, data was excluded pairwise. Data presented as median with interquartile ranges or n (%), as appropriate. EUS—endoscopic ultrasound; ERCP—endoscopic retrograde cholangiopancreatography; US—ultrasound.

**Table 3:** Incidence of American Society for Gastrointestinal Endoscopy 2019 and European Society of Gastrointestinal Endoscopy predictors for detection of common bile duct stones in acute biliary pancreatitis.

	Common Bile Duct Stone		*p*-Value
	Yes (N = 29)	No (N = 57)	
Common bile duct stone visualized on transabdominal ultrasound	4 (100%)	0 (0%)	0.014
Clinical ascending cholangitis (ASGE)/Features of cholangitis (ESGE) ^1^	8 (100%)	0 (0%)	<0.001
Dilated common bile duct on abdominal ultrasound plus elevated bilirubin > 68.4 µmol/L	8 (61.5%)	5 (38.5%)	0.060
Abnormal liver biochemical tests (ASGE)/Abnormal liver function tests (ESGE) ^1^	29 (35.8%)	52 (64.2%)	0.294
Age over 55	22 (38.6%)	35 (61.4%)	0.180
Dilated common bile duct on abdominal ultrasound	16 (59.3%)	11 (40.7%)	0.002

^1^ Terminology by American Society for Gastrointestinal Endoscopy 2019 and European Society of Gastrointestinal Endoscopy 2019 original papers; Data presented as number of patients and percentage. Multiple predictors can be present in each patient. ASGE—American society for gastrointestinal endoscopy; ESGE—European society of gastrointestinal endoscopy.

**Table 4:** Comparison of American Society for Gastrointestinal Endoscopy 2019 probability and European Society of Gastrointestinal Endoscopy likelihood groups for common bile duct stones in acute biliary pancreatitis.

	Common Bile Duct Stone		*p*-Value
	Yes (N = 29)	No (N = 57)	
ASGE high probability	14 (73.7%)	5 (26.3%)	<0.001
ASGE intermediate probability	15 (22.4%)	52 (77.6%)	
ASGE low probability	0	0	
ESGE high likelihood	10 (100%)	0 (0%)	<0.001
ESGE intermediate likelihood	19 (26.8%)	52 (73.2%)	
ESGE low likelihood	0 (0%)	5 (100%)	

Data presented as number of patients and percentage. Each patient was only assigned to one tier for each guideline; if several criteria were met by more than one tier, the highest tier was taken. Pairwise comparisons of ASGE and ESGE high versus intermediate and ESGE high versus low group reached significance after Bonferroni correction for multiple testing. Other pairwise comparisons did not reach significance.

**Table 5:** Sensitivity and specificity of American Society for Gastrointestinal Endoscopy 2019 and European Society of Gastrointestinal Endoscopy algorithms and single predictors for prediction of choledocholithiasis in acute biliary pancreatitis.

	Sensitivity (%)	Specificity (%)
ASGE high probability (any predictor present)	48.3	91.2
ASGE intermediate probability (any predictor present)	100	0
ESGE high likelihood (any predictor present)	34.5	100
ESGE intermediate likelihood (any predictor present)	100	8.8
Common bile duct stone on abdominal ultrasound	13.8	100
Clinical ascending cholangitis (ASGE)/features of cholangitis (ESGE) ^1^	27.6	100
Dilated common bile duct on abdominal ultrasound plus elevated bilirubin oved 68.4 µmol/mL	27.6	91.2
Abnormal liver biochemical tests (ASGE)/abnormal liver function tests (ESGE) ^1^	100	8.8
Age over 55 years	75.9	38.6
Dilated common bile duct on abdominal ultrasound	55.2	80.7

^1^ Terminology by American Society for Gastrointestinal Endoscopy 2019 probability and European Society of Gastrointestinal Endoscopy original papers. ASGE—American society for Gastrointestinal Endoscopy; ESGE—European society of Gastrointestinal Endoscopy.

**Table 6:** Liver biochemistry in patients with and without choledocholithiasis.

	Common Bile Duct Stone		
	Yes (N = 29)	No (N = 57)	*p*-Value
Total bilirubin at admission (µmol/L)	50.0 (27.5–87.5)	42.0 (27.0–73.5)	0.441
Direct bilirubin upon admission (µmol/L)	35.0 (15.5–66.5)	27.0 (11.0–43.0)	0.131
Alkaline phosphatase upon admission (µkat/L)	3.9 (2.3–6.3)	2.2 (1.7–3.6)	0.004
Aspartate aminotransferase upon admission (µkat/L)	4.1 (2.4–7.2)	4.7 (2.2–7.1)	0.817
Alanine aminotransferase upon admission (µkat/L)	4.6 (2.5–9.1)	7.4 (3.2–9.8)	0.379

Data was excluded pairwise in case of missing data. Data are expressed as medians with interquartile ranges.

## Data Availability

Data used for this report are available upon request from the corresponding author.
